# Recommended 24-h movement behavior patterns and equivalent impacts on mental health among Chinese adolescents: a compositional data analysis

**DOI:** 10.3389/fpsyg.2026.1936732

**Published:** 2026-09-09

**Authors:** Yunuo Li, Shengxia Ma, Linli Gong, Hao Yu, Jiaxin Feng

**Affiliations:** College of Physical Education, Sichuan Normal University, Chengdu, China

**Keywords:** physical activity, sedentary behavior, sleep, compositional data analysis, psychological wellbeing, anxiety

## Abstract

**Objective:**

To investigate an optimal 24-h movement behavior recommendation framework to promote mental health and evaluate its equivalent impacts on these psychological outcomes.

**Methods:**

A sample of 319 junior high school students (mean age: 13.73 years, 52.6% male) from Chengdu, China, were included in the study. Their 24-h movement behaviors, comprising light physical activity (LPA), moderate-to-vigorous physical activity (MVPA), sedentary behavior (SB), and sleep (SLP), were objectively measured using accelerometers combined with sleep logs. Psychological wellbeing and anxiety were assessed using the Adolescent Wellbeing Scale and the Chinese version of the Multidimensional Anxiety Scale for Children (MASC-C), respectively. Compositional linear regression, the optimal time-use zone analysis, time reallocation analysis, and equivalent impact curves were applied to systematically examine the relationships between 24-h movement behaviors and mental health outcome.

**Results:**

The overall 24-h movement behavior composition was significantly associated with both psychological wellbeing and anxiety scores. Relative to the remaining behaviors, LPA (β = 6.109, *P* = 0.028) and SLP (β = 18.610, *P* = 0.004) were positively associated with psychological wellbeing, whereas SB (β = −24.476, *P* < 0.001) was negatively associated with psychological wellbeing. For anxiety, SB (β = 32.000, *P* < 0.001) was positively associated with anxiety scores, whereas MVPA (β = −6.353, *P* = 0.029) and SLP (β = −25.827, *P* = 0.009) were negatively associated with anxiety scores. To optimize overall mental health, the recommended durations and ranges for 24-h movement behaviors were 316 min/day (270–420 min/day) for LPA, 68 min/day (50–110 min/day) for MVPA, 510 min/day (460–550 min/day) for SB, and 545 min/day (470–610 min/day) for SLP. Under the one-to-three isoproportional substitution model, three compositional time-reallocation patterns were estimated to correspond to a predicted 0.1-standard deviation (SD) higher psychological wellbeing score: a relative increase in LPA (+ 56.5 min/day), a relative decrease in SB (−26.2 min/day), or a relative increase in SLP (+ 31.8 min/day). Under the one-to-one isotemporal substitution model, a comparable predicted difference was observed when reallocating 27.9 min/day of SB to LPA or 24.7 min/day of SB to SLP. For anxiety, the corresponding one-to-three isoproportional substitution patterns associated with a predicted 0.1-SD lower anxiety score were a relative increase in MVPA (+ 19.4 min/day), a relative decrease in SB (−28.5 min/day), or a relative increase in SLP (+ 32.6 min/day). Similarly, under the one-to-one isotemporal substitution model, a comparable predicted reduction was estimated when reallocating 26.1 min/day of SB to SLP.

**Conclusion:**

For junior high school students, the recommended daily durations of 24-h movement behaviors for overall mental health were316 min of LPA, 68 min of MVPA, and 545 min of SLP, while SB should be limited to no more than 550 min per day. Beyond these specific durations, comparable predicted mental health profiles may correspond to multiple behavioral time-reallocation patterns. In particular, relative reductions in SB showed the strongest magnitude of association with favorable mental health outcomes.

## 1 Introduction

Poor mental health and the rising prevalence of psychiatric disorders have emerged as major public health challenges across the lifespan. Globally, approximately 1 in 12 children and adolescents has a mental disorder (Piao et al., 2022). In China, a national psychiatric epidemiological survey reported a similarly high prevalence of 17.5% in this population (Li et al., 2022). Evidence also indicates that psychological wellbeing tends to decline across the life course (Thygesen et al., 2021; Chen Y. et al., 2022). From a life-course perspective, health and development during childhood and adolescence shape wellbeing in adulthood and may ultimately impact subsequent generations. Importantly, healthier and more sustainable 24-h movement behaviors are consistently associated with better mental health statuses (Groves et al., 2024). In response, Canada released the first integrated 24-Hour Movement Guidelines in 2016 (Tremblay et al., 2016). Subsequently, several countries and regions released similar 24-h movement guidelines (Chen S. et al., 2022; Loo et al., 2022; Okely et al., 2022). Extant evidence suggests that meeting a greater number of these movement recommendations is associated with meaningful psychological benefits, particularly reductions in depressive symptoms (Wilhite et al., 2023; Sun et al., 2024; Wu et al., 2024).

However, the formulation of these guidelines has primarily relied on the evidence for the independent relationships between individual behaviors and health outcomes, supplemented by expert consensus (Bourke et al., 2025). Consequently, they are not fully grounded in a holistic, closed 24-h movement framework and may overlook the inherent covariation among behaviors. Specifically, 24-h movement behaviors include light-intensity physical activity (LPA), moderate-to-vigorous physical activity (MVPA), sedentary behavior (SB), and sleep (SLP), which are constrained to a constant sum of 1,440 min/day and therefore constitute compositional data (Pedišić et al., 2017). Any reallocation of time to one behavior necessarily produces a compensatory change in one or more of the remaining behaviors, making these components highly collinear. Thus, rather than examining isolated behaviors independently, it is necessary to adopt a holistic perspective that treats the 24-h movement composition as an integrated system. To this end, emerging studies have applied compositional data analysis (CoDA) to examine the associations between 24-h movement compositions and mental health outcomes (Huang and Li, 2025; Nyam et al., 2025). By utilizing isometric log-ratio (ilr) transformations, this approach addresses the constant-sum constraint of 24-h time-use data and eliminates multicollinearity among components (Pedišić et al., 2017; Dumuid et al., 2018). Building upon this, Dumuid et al. (2020) developed the “optimal time balance method”, which can mathematically identify optimal movement behavior durations and ranges to maximize physical and mental health (Dumuid et al., 2020). A growing body of research has used this approach to establish tailored optimal time-use balances for children and adolescents in various countries (Dumuid et al., 2020, 2021; Fairclough et al., 2023; Murphy et al., 2025). Nevertheless, empirical evidence on optimal 24-h movement behavior recommendations specifically tailored to mental health promotion among Chinese adolescents remains limited.

Globally, compliance with 24-h movement guidelines among children and adolescents remains persistently low (Hossian et al., 2025). In China, the proportion of youth who simultaneously meet all recommended guidelines ranges from only 0.3% to 26.1% (Huang et al., 2024). Although these guidelines provide valuable time-allocation benchmarks for health promotion, strict adherence to all recommendations is difficult to achieve within the constraints of students' daily lives (Huang et al., 2024; Hossian et al., 2025). This challenge highlights the need for more flexible and adaptive optimization strategies for 24-h movement behaviors. To date, most studies have focused on the direct effects of reallocating time among movement behaviors on psychological wellbeing; for example, replacing SB with LPA or MVPA has been shown to reduce adolescent anxiety scores (Wang et al., 2025). However, identical improvements in health outcomes can potentially be achieved through distinct time-allocation patterns (Dumuid et al., 2022a), a concept referred to as the equivalent impact of 24-h movement behaviors (Ng et al., 2021). Identifying multiple time-use configurations that yield comparable psychological benefits allows youth to select activity profiles that align with their preferences, needs, and practical constraints, thereby improving the flexibility and versatility of lifestyle interventions (Ng et al., 2021; Dumuid et al., 2022a). Compared with rigid, one-size-fits-all guidelines, an equivalent- impact framework may enhance individual autonomy and agency, both of which are important for sustaining behavior change after formal interventions end (Kwasnicka et al., 2016). Previous studies have examined equivalent impacts in relation to adolescent obesity, academic performance, and health-related quality of life (Talarico and Janssen, 2018; Ng et al., 2021; Dumuid et al., 2022a). For example, a 0.1-unit increase in the BMI z-score was associated with an additional 23 min of LPA, a 13-min reduction in MVPA, an additional 89 min of SB, or a 65-min reduction in SLP, with each estimate evaluated relative to the remaining behaviors and based on the sample compositional mean (Talarico and Janssen, 2018). However, few studies have examined the equivalent impact of 24-h movement behaviors on the mental health of children and adolescents.

To address these research gaps, this study utilized CoDA to systematically examine the associations between 24-h movement behaviors and mental health outcomes among Chinese adolescents. The specific objectives were to: (1) apply the optimal time balance method to establish 24-h movement behavior recommendations, including optimal durations and ranges, for promoting mental health; and (2) simulate equivalent impact across different time-reallocation scenarios to identify multiple time-allocation patterns that yield comparable psychological benefits. Ultimately, these empirical findings aim to provide flexible, actionable evidence to inform school-based physical activity interventions and youth public health policies in China.

## 2 Materials and methods

### 2.1 Participants

Between September 2025 and June 2026, a total of 450 junior high school students were recruited from four schools in Chengdu, China. Prior to the assessments, written informed consent was obtained from all participating students and their parents or legal guardians. Participants were informed that they could voluntarily withdraw from the study at any stage without penalty. The exclusion criteria were as follows: (1) restricted physical activity for any reason; (2) failure to wear the accelerometer as instructed or incomplete sleep logs, resulting in invalid 24-h movement behavior data; (3) missing or unmatchable basic socio-demographic information; and (4) severe somatic or psychiatric disorders. The study protocol was reviewed and approved by the Ethics Committee of Sichuan Normal University (Approval No.: 2026LS0126). Of the 450 recruited participants, 75 were excluded due to insufficient valid accelerometer wear days, 35 due to incomplete sleep logs, and 21 due to missing or unmatched demographic information. After screening, a final valid sample of 319 participants (168 boys and 151 girls) was included in the statistical analysis.

The sample size for this study was estimated *a priori* using G^*^Power software (version 3.1.9.7). Given that the statistical analysis involved a multivariable linear regression model based on isometric log-ratio (ilr) coordinates, statistical power was calculated via F tests within the Linear multiple regression: Fixed model, *R*^2^ deviation from zero framework. Assuming a medium effect size (*f*^2^ = 0.15), a significance level (α = 0.05), a target statistical power (1—β) of 0.95, the power analysis indicated that a minimum total sample size of 172 participants was required. The final sample size achieved in this study (*N* = 319) exceeded this threshold, ensuring sufficient statistical power for the composition-based regression models.

### 2.2 Methods

#### 2.2.1 24h movement behaviors

The 24-h movement behaviors were objectively measured using ActiGraph wGT3X-BT accelerometers. Before data collection, researchers provided standardized instructions on wearing requirements and precautions. Participants were asked to wear the accelerometer on their right hip for 7 consecutive days, and to remove it only during water-based activities (e.g., swimming). Researchers checked compliance once daily at noon on school days and reminded participants to complete the sleep log. Data were considered valid if a participant had at least 4 valid days within 1 week, including 3 weekdays and 1 weekend day, with at least 10 h of waking wear time per day (Tudor-Locke et al., 2015). Physical activity intensity and SB were classified using the Evenson et al. cut-points: 0–100 counts/min for SB, 101–2295 counts/min for LPA, 2296–4011 counts/min for moderate physical activity, and ≥ 4012 counts/min for vigorous physical activity (Evenson et al., 2008). Non-wear time was defined as any continuous interval of at least 60 min with consecutive zero counts per minute (CPM) (Troiano et al., 2008). MVPA duration was calculated as the sum of moderate and vigorous physical activity. In addition, sleep logs were used to record precise bedtimes and wake-up times during the testing period; these were cross-validated using ActiLife software to estimate daily sleep duration. Finally, the daily durations of MVPA, LPA, SB, and SLP were utilized as the movement behavior composition data for analysis.

#### 2.2.2 Psychological wellbeing

The EPOCH measure, comprising Engagement, Perseverance, Optimism, Connectedness, and Happiness, was employed to assess psychological wellbeing among junior high school students (Kern et al., 2016). The Chinese version of this scale has been validated in a large sample of Chinese adolescents, demonstrating a robust factor structure and high internal consistency, and is therefore a reliable instrument for assessing positive psychological functioning in youth (Zeng and Kern, 2019). The scale includes 20 items across five core positive psychological characteristics. Items are rated on a 5-point Likert scale ranging from 1 (not at all like me) to 5 (very much like me), yielding a total score range of 20–100. Higher scores indicate greater positive psychological functioning and psychological wellbeing. In the present study, the Cronbach's α coefficients for the five dimensions ranged from 0.790 to 0.935, and the Cronbach's α for the overall EPOCH score was 0.934.

#### 2.2.3 Anxiety

The Chinese revised version of the Multidimensional Anxiety Scale for Children (MASC-C) was used to assess anxiety levels among junior high school students (Yao et al., 2007). Previous research has supported the reliability and validity of this scale among Chinese middle school students, reporting an overall Cronbach's α coefficient of 0.91 and a 1-month test-retest reliability (intraclass correlation coefficient) of 0.84. Confirmatory factor analysis also supported the original four-factor structure, measurement invariance across genders and ages, and good internal consistency and structural validity, indicating that the scale is an effective tool for evaluating anxiety symptoms in Chinese adolescents (Yao et al., 2007). The MASC consists of 39 items across four dimensions: physical symptoms (12 items), social anxiety (9 items), separation anxiety (9 items), and harm avoidance (9 items). It assesses anxiety symptom severity during the past week. Items are scored on a 4-point Likert scale ranging from 0 (never) to 3 (always), yielding a total score range of 0–117, with higher scores indicating more severe anxiety symptoms. In the present study, the Cronbach's α coefficients for the four dimensions ranged from 0.883 to 0.928, and the Cronbach's α for the overall MASC-C score was 0.955.

#### 2.2.4 Covariates

A socio-demographic questionnaire was used to collect information on students' gender, age, grade, body mass index (BMI), boarding status, parental education level, and household income. Parental education level was categorized according to whether parents had received formal higher education, with a postgraduate degree or above as the highest category. These socio-demographic factors were included as covariates in subsequent statistical analyses.

#### 2.2.5 Statistical analysis

Statistical analyses were conducted according to the CoDA guidelines proposed by Dumuid et al. (2018) and the optimal time-use zone analysis method developed by Dumuid et al. (2020). Data processing and statistical modeling were performed in R software (version 4.5.2) using the “compositions,” “zCompositions,” and “devtools” packages. During preprocessing, no zero values were identified in any of the four movement behavior components (LPA, MVPA, SB, and SLP). Participants' demographic characteristics and health outcomes were expressed as means ± standard deviations (SD) for continuous variables and as frequencies and percentages (n, %) for categorical variables. The central tendency of the 24-h movement behavior data was characterized using compositional geometric means, and dispersion was described using a log-ratio variation matrix. In this matrix, larger log-ratio variance indicates weaker proportional co-dependence between a pair of movement behaviors, whereas a smaller variance indicates stronger proportional co-dependence.

##### 2.2.5.1 Models examining the relationships between 24-h movement behaviors and mental health indicators

Compositional multiple linear regression models were constructed to examine the associations between 24-h movement behaviors and mental health outcomes. The independent variables were the ilr-transformed 24-h movement behavior data. Specifically, three *ilr* coordinates were generated from the four-part time-use composition. By sequentially rearranging the components so that each movement behavior appeared once in the first position, the corresponding first ilr coordinate was obtained, allowing the relative effect of each individual behavior to be interpreted against the remaining three behaviors. The dependent variables were psychological wellbeing scores and anxiety scores. Socio-demographic factors, including age, gender, BMI, grade, boarding status, parental education level, and household income, were adjusted for as covariates. Statistical significance was set at *P* < 0.05. Throughout the model building, diagnostic evaluations were conducted for all fitted models to assess linearity, residual normality, homoscedasticity, and potential outliers.

##### 2.2.5.2 Optimization of 24-h movement behavior recommendations for maximizing psychological wellbeing and minimizing anxiety

To improve the predictive performance, quadratic terms of the coordinates (ilr^2^) were added to the baseline linear regression models. The quadratic models were compared with the corresponding linear models using analysis of variance (ANOVA), and quadratic terms were retained in the final predictive models when they significantly improved model fit (*P* < 0.05). The overall *F*-statistic for each model was also evaluated by ANOVA to determine whether there was a significant omnibus association between the *ilr*-transformed 24-h movement behavior compositions and each mental health outcome. A predictive grid of hypothetical 24-h time-use compositions was generated based on the empirical distribution of the sample. The outer boundaries of this multi-dimensional grid were restricted to values within the sample mean plus or minus three standard deviations (± 3SD) for each behavior to avoid unrealistic extrapolation beyond the observed data range. Within these empirical boundaries, a loop function in R was used to simulate all mathematically possible 24-h movement behavior combinations in 10-min increments, with each composition constrained to the constant sum of 1,440 min/day. The selected optimal predictive models were then used to estimate the psychological wellbeing and anxiety scores for every time-use configuration within the generated grid. Finally, predicted psychological wellbeing scores were ranked from highest to lowest, and the predicted anxiety scores were ranked from lowest to highest. The 24-h movement behavior compositions corresponding to the top 5% of the predicted outcomes, defined as the optimal outcome zone, were extracted. The compositional geometric means and ranges of these top-performing profiles were then calculated to establish optimal 24-h movement behavior recommendations for psychological wellbeing and anxiety, respectively. The overlapping region of the 24-h movement behavior combinations that simultaneously corresponded to the top 5% of psychological wellbeing scores and the lowest 5% of anxiety scores was identified as the overall best mental health zone. The compositional geometric means and ranges of the four movement behaviors within this intersection were then calculated.

##### 2.2.5.3 Associations between 24-h movement behavior time reallocation and mental health scores

Two time-reallocation models were applied to estimate the predictive effects of behavioral time reallocation on psychological wellbeing and anxiety scores: one-to-one isotemporal substitution and one-to-many isoproportional substitution (Zhang et al., 2022). The sample compositional mean was used as the reference composition, with its baseline *ilr* coordinates denoted as:


$$\begin{array}{l} ilr_{mean}=ilr\left[LPA_{mean},MVPA_{mean},SB_{mean},SLP_{mean}\right] \end{array}$$

In the one-to-one isotemporal substitution model, a fixed duration (e.g., 15min/day) was reallocated from one source behavior to a target behavior, keeping the remaining two behaviors constant. For instance, replacing 15min/day of SB with LPA yielded new coordinates:


$$ilr_{new}=ilr\left[LPA_{mean}+15\;\min,MVPA_{mean},SB_{mean}-15\;\min,SLP_{mean}\right]$$

In the one-to-many isoproportional substitution model, when the duration of a target behavior *X*_*k*_ was changed by Δ*X*_*k*_ (e.g., a 10% shift of its compositional mean), the remaining three non-target behaviors adjusted proportionally to maintain the 24-h time budget (1440 min/day). The updated coordinates were expressed as:


$$ilr_{new}=ilr\left[LPA_{mean}+\Delta X_{k},MVPA_{mean}\times \left(1-S\right),SB_{mean}\times \left(1-S\right),SLP_{mean}\times \left(1-S\right)\right]$$

where *S* is the compensation factor calculated as:


$$\begin{array}{l} S=\frac{\Delta X_{k}}{1440-X_{k}^{mean}} \end{array}$$

For both substitution models, the updated *ilr*_*new*_ coordinates were entered into the fitted compositional linear regression models to calculate the estimated score changes relative to baseline ($\Delta \hat{Y}={\hat{Y}}_{new}-{\hat{Y}}_{mean}$) along with 95% confidence intervals.

##### 2.2.5.4 Construction of equivalent impact curves

**Figure 1:**
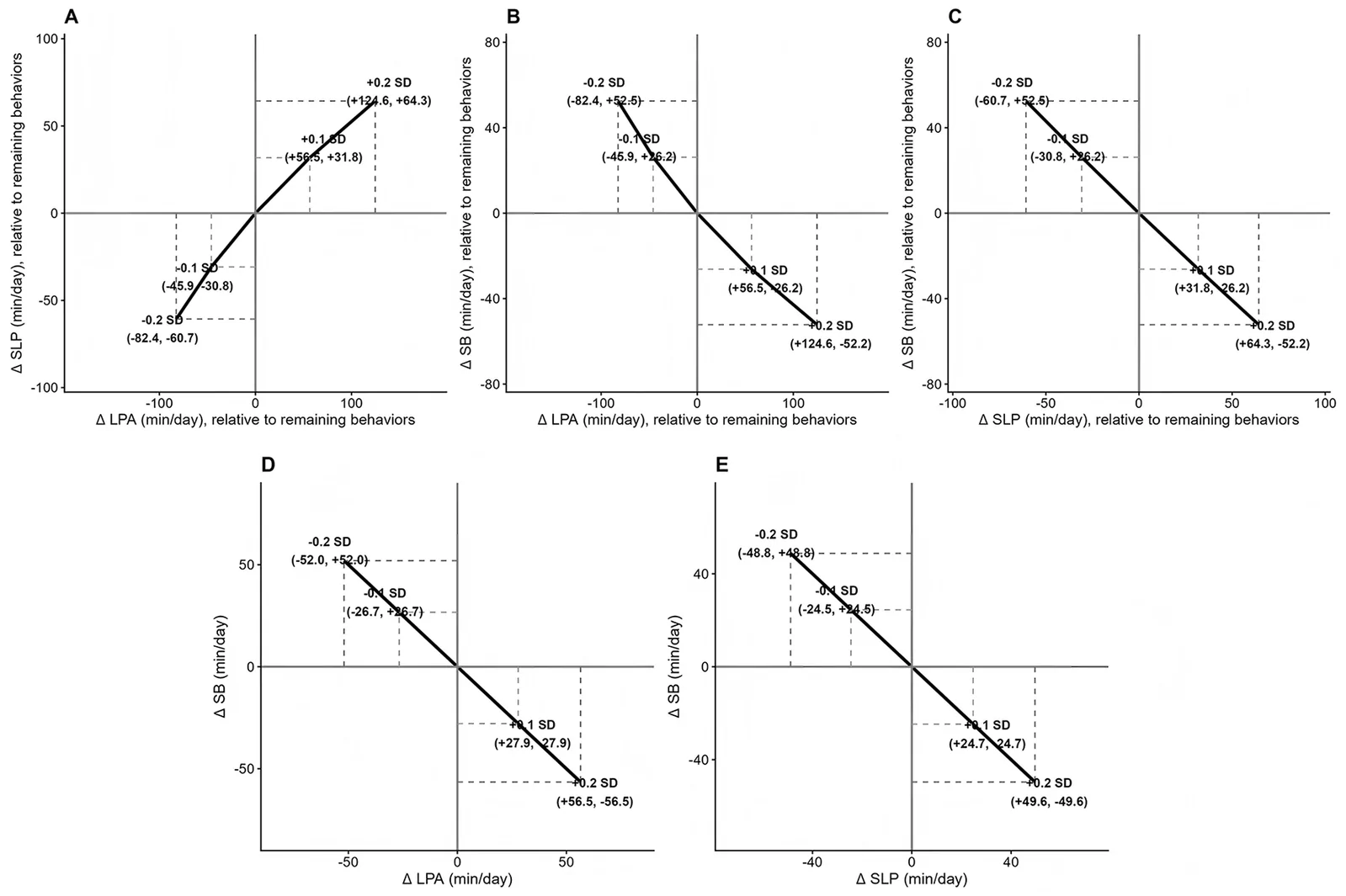
Equivalent impact curves of 24-h movement behaviors on psychological wellbeing scores. Panels **(A–C)** show the “one-to-three” isoproportional time reallocation, where the solid black line represents time-use compositions yielding an equivalent change in the psychological wellbeing score. The dashed lines and coordinates indicate the required proportional reallocation time to achieve a ± 0.1 and ±0.2 SD change in wellbeing. For example, in Panel **(A)**, “+0.1 SD (+56.5, +31.8)” indicates that a proportional increase of 56.5 min/day in LPA and 31.8 min/day in SLP (while proportionally decreasing the remaining behaviors) is associated with a 0.1 SD increase in the psychological wellbeing score. Panels **(D, E)** represent the “one-to-one” isotemporal substitution, showing the linear trajectory of replacing one behavior with another while holding others constant. For instance, in Panel **(D)**, “+ 0.1 SD (+ 27.9, −27.9)” indicates that replacing 27.9 min/day of SB with an equivalent duration of LPA is associated with a 0.1 SD increase in the psychological wellbeing score.

Equivalent impact curves (iso-effect contours) were constructed to visually illustrate behavioral time-reallocation configurations associated with comparable predicted mental health outcomes (Ng et al., 2021). Using predefined standardized score differences of ±0.1 and ±0.2 SD in psychological wellbeing and anxiety scores, the corresponding changes in movement behavior durations were estimated from the fitted compositional regression models and plotted pairwise. These thresholds were selected with reference to existing compositional time-use equivalence studies (Ng et al., 2021; Talarico and Janssen, 2018). Effect sizes of 0.1–0.2 SD correspond to daily time reallocations of several tens of minutes, which are practically feasible for adolescents in daily life contexts. Under one-to-many isoproportional substitution, equivalent impact curves characterize the substitution trade-offs among movement behaviors when each behavior undergoes proportional reallocation to reach the identical target effect size. The corresponding duration changes of two behaviors achieving the same target effect size were paired and plotted in the equivalent impact curves. For example, in Figure 1A, a +0.1SD higher psychological wellbeing score corresponded to proportionally increasing LPA by 56.5 min/day or proportionally increasing SLP by 31.8min/day (both relative to the remaining behaviors), yielding the paired coordinate (+56.5, + 31.8). Under one-to-one isotemporal substitution, equivalent impact curves characterize direct time exchanges between two specific behaviors while keeping remaining behaviors constant. The duration changes of the target and source behaviors required to reach the identical target effect size were paired and plotted in the equivalent impact curves. For example, replacing 27.9 min/day of SB with LPA achieved a +0.1 SD higher wellbeing score, yielding the paired coordinate (+ 27.9, −27.9). For full transparency and reproducibility, the complete R scripts used for all analyses are publicly available in the Open Science Framework repository (https://osf.io/24cqa/).

## 3 Results

### 3.1 Descriptive statistics

A total of 319 junior high school students were included in the final analysis. The mean age was 13.73 ± 0.82 years, and the mean BMI was 19.82 ± 3.36 kg/m^2^. The sample comprised 168 boys (52.6%) and 151 girls (47.3%). Regarding grade distribution, 97 students were in grade 7 (30.4%), 80 in grade 8 (25.0%), and 142 in grade 9 (44.5%). For the mental health outcomes, the mean psychological wellbeing score was 74.17 ± 15.44, and the mean anxiety score was 33.87 ± 21.97. The compositional geometric means and relative daily time proportions of the 24-h movement behaviors were as follows: LPA, 203.22 min/day (14.11%); MVPA, 41.14 min/day (2.86%); SB, 695.34 min/day (48.29%) and SLP, 500.29 min/day (34.74%) (Table 1).

**Table 1:** Descriptive statistics of middle school students.

Variables	*N* = 319
Age, (year), mean (SD)	13.73 ± 0.82
BMI (kg/m^2^, M ± SD)	19.82 ± 3.36
Sex *n* (%)	
Boys	168(52.6%)
Girls	151(47.3%)
Grade, *n* (%)	
Grade 7	97(30.4%)
Grade 8	80(25.0%)
Grade 9	142(44.5%)
Activity behaviors, arithmetic mean (%)	
LPA	213.39(14.82%)
MVPA	46.66(3.24%)
SB	687.52(47.76%)
SLP	492.44(34.20%)
Activity behaviors, composition mean (%)	
LPA	203.22(14.11%)
MVPA	41.14(2.86%)
SB	695.34(48.29%)
SLP	500.29(34.74%)
Psychological wellbeing	74.17 ± 15.44
Anxiety	33.87 ± 21.97

BMI, body mass index; SD, standard deviation; MVPA, moderate-to-vigorous physical activity; LPA, light physical activity; SLP, sleep; SB, sedentary behavior. Both arithmetic means and compositional geometric means are reported. Arithmetic means describe individual behavior durations independently without applying relative constant-sum closure constraints. Compositional geometric means represent the geometric center of the 24-h time-use composition under closure properties, accounting for relative co-dependence among movement behaviors.

The results of the log-ratio variation matrix for the 24-h movement behavior data showed that the pairwise log-ratio variance between SB and SLP was the smallest (0.039), indicating the strongest proportional co-dependence between these two behaviors. This was followed by the log-ratio variance between LPA and SLP (0.180), which also demonstrated a strong relative interdependence. In contrast, the largest pairwise log-ratio variances consistently involved MVPA, suggesting that MVPA accounted for a smaller share of daily time, displaying weaker co-dependence with the other three movement behavior components (Table 2).

**Table 2:** Log-ratio variance matrix of the 24-h movement behavior components.

Movement behavior	LPA	MVPA	SB	SLP
LPA	0	0.407	0.241	0.18
MVPA	0.407	0	0.386	0.302
SB	0.241	0.386	0	0.039
SLP	0.18	0.302	0.039	0

### 3.2 Association between 24-h movement behavior and mental health

Model diagnostics were evaluated using residual plots, Q-Q plots, scale-location plots, and residuals vs. leverage plots. Across all models, no problematic multicollinearity (VIF < 5.0), influential outliers (Cook's distance < 0.50), or residual autocorrelation (*P* > 0.05) were detected. Residuals were approximately normally distributed with no clear non-linear patterns or severe heteroscedasticity, confirming that regression assumptions were satisfied (Supplementary Table 1 and Supplementary Figures 1–2).

The overall 24-h movement behavior composition was significantly associated with both psychological wellbeing (*R*^2^ = 0.264, *P* < 0.001) and anxiety (*R*^2^ = 0.116, *P* < 0.001) (Table 3). LPA was positively associated with psychological wellbeing (β = 6.109, *P* < 0.05), whereas MVPA was negatively associated with anxiety (β = −6.353, *P* < 0.05). SB was negatively associated with psychological wellbeing (β = −24.476, *P* < 0.001) and positively associated with anxiety (β = 32.000, *P* < 0.001). Finally, SLP was positively associated with psychological wellbeing (β = 18.610, *P* < 0.01) and negatively associated with anxiety (β = −25.827, *P* < 0.01).

**Table 3:** Compositional regression analysis of the associations between 24-h movement behaviors and mental health outcomes.

Regression models (ilr)	β	SE	*p*-value	*R* ^2^	Model *p*
**Psychological wellbeing**				0.264	< 0.001
LPA vs. (SLP + SB + MVPA)	6.109	2.773	0.028		
MVPA vs. (SLP + SB + LPA)	−0.243	1.853	0.896		
SB vs. (SLP + LPA + MVPA)	−24.476	4.857	< 0.001		
SLP vs. (SB + LPA + MVPA)	18.610	6.336	0.004		
**Anxiety**				0.116	< 0.001
LPA vs. (SLP + SB + MVPA)	0.180	4.326	0.667		
MVPA vs. (SLP + SB + LPA)	−6.353	2.890	0.029		
SB vs. (SLP + LPA + MVPA)	32.000	7.561	< 0.001		
SLP vs. (SB + LPA + MVPA)	−25.827	9.884	0.009		

The β value represents the estimated regression coefficient of the first ilr coordinate, indicating the direction and magnitude of the association between the relative dominance of one specific target behavior (compared to the geometric mean of the remaining three behaviors) and the mental health outcomes. All models have been adjusted for age, gender, grade, boarding status, BMI, parents' education level, and family income.

### 3.3 Mental health-based recommendations for 24-h movement behaviors

Table 4 presents the compositional means and ranges of the 24-h movement behavior configurations associated with the predicted top 5% and top 10% mental health outcomes. The collective 24-h movement behavior time composition of junior high school students serves as an effective omnibus predictor of their psychological wellbeing and anxiety (F-statistics: both *P* < 0.05). Specifically, the recommended durations and ranges associated with the top 5% highest psychological wellbeing scores were 220 min/day (120–300) for LPA, 86 min/day (50–120) for MVPA, 522 min/day (430–590) for SB, and 612 min/day (550–640) for SLP. The corresponding recommendations associated with the lowest 5% anxiety scores were 388 min/day (310–450) for LPA, 75 min/day (40–120) for MVPA, 455 min/day (420–510) for SB, and 523 min/day (440–600) for SLP.

**Table 4:** Associations between 24-h movement behavior compositions and psychological wellbeing outcomes, and the estimated optimal time-use zones.

Model summary (for ilrs) ^b^			Activity center (range) associated with top 5% and 10% outcomes (min/day)			
	*F*	*P*	LPA	MVPA	SB	SLP
Psychological wellbeing^a^						
5%	6.485	< 0.001	220(120–300)	86(50–120)	522(430–590)	612(550–640)
10%			253(110–450)	77(30–120)	528(420–600)	582(390–640)
Anxiety^a^						
5%	4.792	< 0.001	388(310–450)	75(40–120)	455(420–510)	523(440–600)
10%			367(270–450)	73(40–120)	471(420–550)	529(430–620)
overlapping zone^c^ (min/day)			316(270–420)	68(50–110)	510(460–550)	545(470–610)

^a^The models include additional squared terms for the ilr coordinates to account for non-linear relationships. ANOVA testing confirmed that the quadratic models provided a significantly better fit than the linear models for both psychological wellbeing (*F* = 2.74, *P* = 0.014) and anxiety (*F* = 2.50, *P* = 0.023). ^b^*F*-statistic and p-value indicate the overall significance of the 24-hour movement composition (ILRs) in predicting the respective outcome. ^c^Defined as the compositional center (and range) of the shared overlapping area between the optimal time-use zones. Due to the absence of a mutual intersection at the top 5% threshold, the zone was determined using the intersection of the top 10% optimal time-use compositions for both maximizing psychological wellbeing and minimizing anxiety.

Because no overlap was observed between the recommended behavior ranges for maximizing psychological wellbeing and minimizing anxiety under the top 5% optimal outcome thresholds, a comprehensive optimization scheme was derived by identifying the overlapping region of the top 10% optimal outcomes for both indicators. The balanced optimal 24-h movement behavior recommendation scheme, which simultaneously optimized psychological wellbeing and mitigated anxiety symptoms, consisted of the following durations and target ranges: LPA, 316 min/day (270–420); MVPA, 68 min/day (50–110); SB, 510 min/day (460–550); and SLP, 545 min/day (470–610).

### 3.4 Associations between simulated 24-h movement behavior time reallocation and mental health outcomes

Table 5 presents the estimated changes in predicted psychological wellbeing and anxiety scores under the one-to-one isotemporal substitution framework, in which a fixed duration of 15 min/day was reallocated from one movement behavior to another while the remaining two components were held constant. Reallocating 15 min/day from SB to LPA was associated with a predicted 0.839-point higher psychological wellbeing score (95% CI: 0.503, 1.176) and a predicted 0.593-point lower anxiety score (95% CI: −1.118, −0.068). In contrast, reallocating 15 min/day from MVPA to LPA was associated with a predicted 2.506-point higher anxiety score (95% CI: 0.085, 4.927). Reallocating 15 min/day from SB to MVPA was associated with a predicted 2.315-point lower anxiety score (95% CI: −3.856, −0.773). Conversely, reallocating 15 min/day from LPA to SB was associated with a predicted 0.858-point lower psychological wellbeing score (95% CI: −1.216, −0.500) and a predicted 0.580-point higher anxiety score (95% CI: 0.021, 1.138). Reallocating 15 min/day from SLP to SB was associated with a predicted 0.943-point lower psychological wellbeing score (95% CI: −1.436, −0.450) and a predicted 1.272-point higher anxiety score (95% CI: 0.503, 2.042), whereas reallocating 15 min/day from MVPA to SB was associated with a predicted 3.086-point higher anxiety score (95% CI: 0.850, 5.322). Finally, reallocating 15 min/day from SB to SLP was associated with a predicted 0.938-point higher psychological wellbeing score (95% CI: 0.451, 1.426) and a predicted 1.265-point lower anxiety score (95% CI: −2.025, −0.505), whereas reallocating 15 min/day from MVPA to SLP was associated with a predicted 1.834-point higher anxiety score (95% CI: 0.559, 4.227).

**Table 5:** Estimated changes in psychological wellbeing and anxiety scores following a 15-min one-to-one isotemporal substitution of 24-h movement behaviors.

Outcome	Predicted score changes				
	Component	LPA↓	MVPA↓	SB↓	SLP↓
Psychological wellbeing	LPA↑	—	0.472 (−1.080, 2.024)	0.839 (0.503, 1.176)^*^	−0.114 (−0.703, 0.475)
	MVPA↑	−0.471 (−1.600, 0.658)	—	0.397 (−0.592, 1.385)	−0.556 (−1.658, 0.546)
	SB↑	−0.858 (−1.216, −0.500)^*^	−0.357 (−1.790, 1.076)	—	−0.943 (−1.436,−0.450)^*^
	SLP↑	0.070 (−0.533, 0.674)	0.572 (−0.962, 2.106)	0.938 (0.451, 1.426)^*^	—
Anxiety	LPA↑	—	2.506 (0.085, 4.927)^*^	−0.593 (−1.118, −0.068)^*^	0.692 (−0.227, 1.611)
	MVPA↑	−1.722 (−3.484, 0.040)	—	−2.315 (−3.856, −0.773)^*^	−1.029 (−2.748, 0.689)
	SB↑	0.580 (0.021, 1.138)^*^	3.086 (0.850, 5.322)^*^	—	1.272 (0.503, 2.042)^*^
	SLP↑	−0.673 (−1.614, 0.269)	1.834 (0.559, 4.227)^*^	−1.265 (-2.025, −0.505)^*^	—

Data are presented as the estimated changes in predictive scores with 95% CIs. Row labels with an upward arrow (↑) indicate the behavior being increased, while column labels with a downward arrow (↓)indicate the behavior being replaced (decreased) by exactly 15 min per day, with the remaining two behaviors kept constant. ^*^P < 0.05.

Table 6 reports the predicted changes in psychological wellbeing and anxiety scores under the one-to-many isoproportional substitution framework. This model estimated score differences when the mean duration of a target movement behavior was increased or decreased by 10%, with the corresponding time proportionally reallocated from or to the remaining three components. A 10% increase in LPA (20.32 min/day), with proportional reallocation from the other behaviors, was associated with a predicted 0.592-point higher psychological wellbeing score (95% CI: 0.063, 1.121), whereas a 10% decrease in LPA was associated with a predicted 0.644-point lower psychological wellbeing score (95% CI: −1.219, −0.069). A 10% increase in MVPA (4.11 min/day) was associated with a predicted 0.541-point lower anxiety score (95% CI: −1.024, −0.057), whereas a 10% decrease in MVPA was associated with a predicted 0.596-point higher anxiety score (95% CI: 0.062, 1.129). A 10% increase in SB (69.53 min/day) was associated with a predicted 4.098-point lower psychological wellbeing score (95% CI: −5.695, −2.501) and a predicted 5.358-point higher anxiety score (95% CI: 2.220, 8.494). Conversely, a 10% decrease in SB was associated with a predicted 4.126-point higher psychological wellbeing score (95% CI: 2.518, 5.733) and a predicted 5.394-point lower anxiety score (95% CI: −7.902, −2.886). Finally, a 10% increase in SLP (50.03 min/day) was associated with a predicted 2.418-point higher psychological wellbeing score (95% CI: 0.798, 4.038) and a predicted 3.355-point lower anxiety score (95% CI: −5.882, −0.828), whereas a 10% decrease in SLP was associated with a predicted 2.534-point lower psychological wellbeing score (95% CI: −4.232, −0.836) and a predicted 3.517-point higher anxiety score (95% CI: 0.868, 6.165).

**Table 6:** The effects of a 10% proportional one-to-three reallocation of 24-hour movement behaviors on mental health outcomes.

Outcome	Time	LPA	MVPA	SB	SLP
Psychological wellbeing	+ 10%	0.592 (0.063, 1.121) ^*^	−0.021 (−0.331, 0.290)	−4.098 (−5.695, −2.501)^*^	2.418 (0.798, 4.038)^*^
	−10%	−0.644 (−1.219, −0.069) ^*^	0.023 (−0.319, 0.365)	4.126 (2.518, 5.733)^*^	−2.534 (−4.232, −0.836)^*^
Anxiety	+ 10%	0.017 (−0.807, 0.842)	−0.541 (−1.024, −0.057)^*^	5.358 (2.220, 8.494)^*^	−3.355 (−5.882,−0.828)^*^
	−10%	−0.019 (−0.916, 0.878)	0.596 (0.062, 1.129)^*^	−5.394 (−7.902, −2.886)^*^	3.517 (0.868, 6.165)^*^

Data are presented as the estimated change in predictive scores with 95% CIs. Reallocation (+ 10% / −10%) represents a change equivalent to 10% of the baseline compositional geometric mean of the target behavior, with the remaining three behaviors adjusting proportionally to maintain the 24-h budget. In this study, the 10% allocation unit corresponds to 20.32 min for LPA, 4.11 min for MVPA, 69.53 min for SB, and 50.03 min for SLP, respectively. ^*^P < 0.05.

### 3.5 Equivalent impacts of 24-h movement behavior on mental health outcomes

Figure 1 presents the equivalent impact curves of 24-h movement behaviors for psychological wellbeing. Under the one-to-many substitution framework, compositional patterns corresponding to predicted differences of ± 0.1 and ± 0.2 SD in psychological wellbeing scores were identified. Predicted differences of + 0.1 SD and + 0.2 SD in psychological wellbeing scores corresponded to a relative increase in LPA of 56.5 min/day and 124.6 min/day (Figure 1A), a relative decrease in SB of 26.2 min/day and 52.2 min/day (Figure 1B), or a relative increase in SLP of 31.8 min/day and 64.3 min/day (Figure 1C), respectively. Conversely, predicted reductions of −0.1 SD and −0.2 SD in wellbeing corresponded to a relative decrease in LPA of 45.9 min/day and 82.4 min/day (Figure 1A), a relative increase in SB of 26.2 min/day and 52.5 min/day (Figure 1B), or a relative decrease in SLP of 30.8 min/day and 60.7 min/day (Figure 1C), respectively. Under the one-to-one isotemporal substitution framework, predicted differences of + 0.1 SD and +0.2 SD in psychological wellbeing scores corresponded to reallocating 27.9 min/day and 56.5 min/day of SB to LPA (Figure 1D), or 24.7 min/day and 49.6 min/day of SB to SLP (Figure 1E). In contrast, predicted reductions of −0.1 SD and−0.2 SD in psychological wellbeing were associated with replacing 26.7 min/day and 52.0 min/day of LPA with SB (Figure 1D), or replacing 24.5 min/day and 48.8 min/day of SLP with SB (Figure 1E).

**Figure 2:**
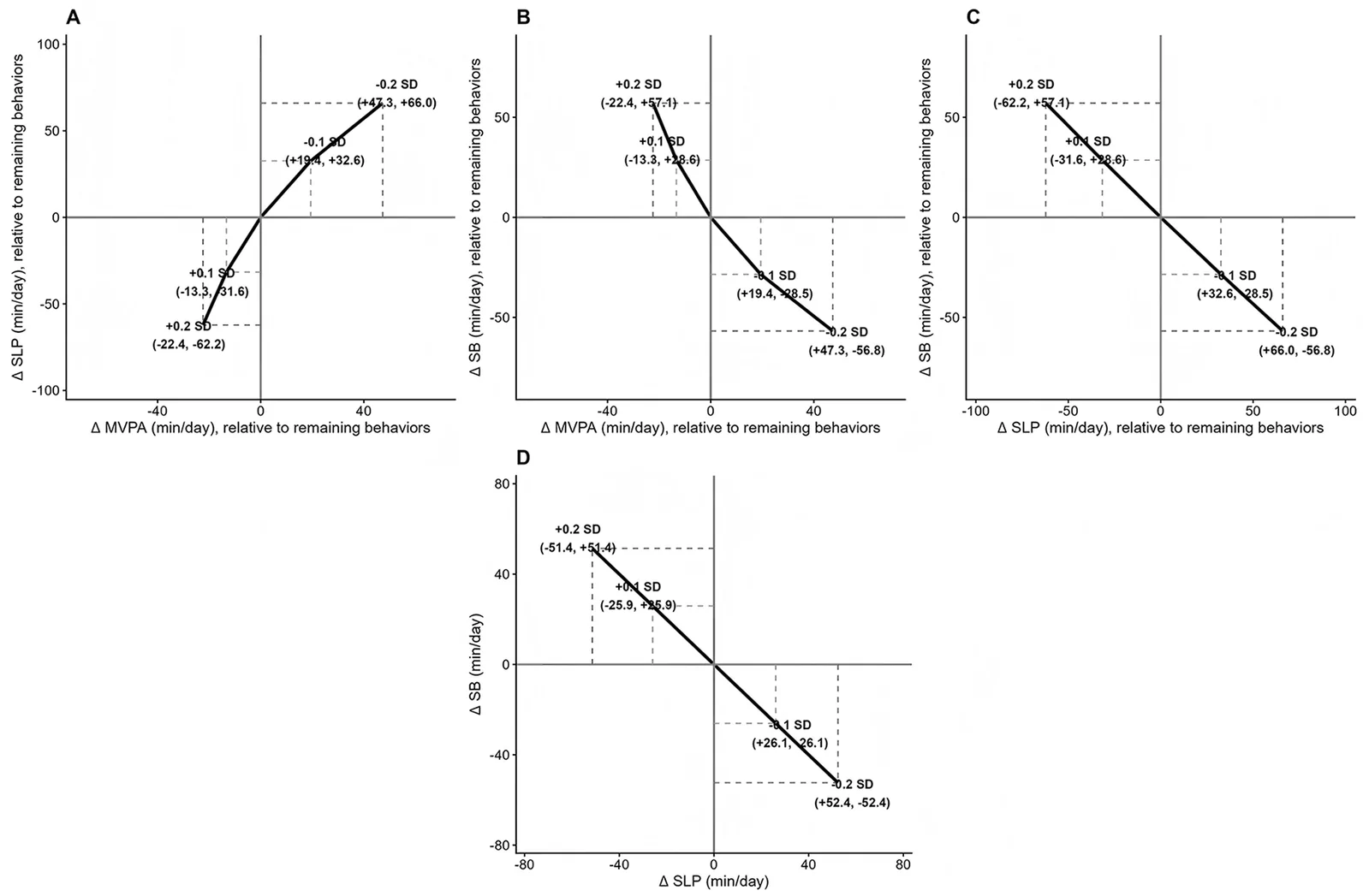
Equivalent impact curves of 24-h movement behaviors on anxiety scores. Panels **(A–C)** illustrate the “one-to-three” isoproportional time reallocation, where the solid black line represents time-use compositions yielding an equivalent change in the total anxiety score. The dashed lines and coordinates indicate the required proportional reallocation time to achieve a ± 0.1 and ± 0.2 SD shift in anxiety. A negative change (e.g., −0.2 SD) reflects an improvement (reduction) in anxiety, while a positive change (e.g., + 0.2 SD) reflects a worsening (elevation) of anxiety. For example, in Panel **(B)**, “−0.2 SD (+ 47.3, −56.8)” indicates that a proportional increase of 47.3 min/day in MVPA, combined with a proportional decrease of 56.8 min/day in SB, is associated with a 0.2 SD reduction in anxiety. Panel **(D)** represents the “one-to-one” isotemporal substitution between sleep (SLP) and sedentary behavior (SB), showing the linear trajectory of direct time exchange while keeping the remaining behaviors constant. For instance, “-0.2 SD (+ 52.4, −52.4)” denotes that replacing 52.4 min/day of SB with an equivalent duration of SLP is estimated to reduce the total anxiety score by 0.2 SD.

Figure 2 illustrates the equivalent impact curves of 24-h movement behaviors for students' anxiety. Under the one-to-many substitution framework, predicted increases of +0.1 SD and +0.2 SD in anxiety, corresponded to a relative decrease in MVPA of 13.3 min/day and 22.4 min/day (Figure 2A), a relative increase in SB of 28.6 min/day and 57.1 min/day (Figure 2B), or a relative decrease in SLP of 31.6 min/day and 62.2 min/day (Figure 2C), respectively. Conversely, predicted reductions of −0.1 SD and −0.2 SD in anxiety scores corresponded to a relative increase in MVPA of 19.4 min/day and 47.3 min/day (Figure 2A), a relative decrease in SB of 28.5 min/day and 56.8 min/day (Figure 2B), or a relative increase in SLP of 32.6 min/day and 66.0 min/day (Figure 2C), respectively. Under the one-to-one isotemporal substitution framework, predicted increases of + 0.1 SD and + 0.2 SD in anxiety were associated with replacing 25.9 min/day and 51.4 min/day of SLP with SB (Figure 2D). In contrast, an equivalent predicted reduction in anxiety of −0.1 SD and −0.2 SD were estimated for replacing 26.1 min/day and 52.4 min/day of SB with SLP (Figure 2D).

## 4 Discussion

### 4.1 Recommended 24-h movement behavior profiles associated with adolescent mental health

Using a compositional data analysis framework, this study identified 24-h movement behavior compositions associated with optimal mental health profiles and evaluated equivalent associations across different time-reallocation scenarios among Chinese junior high school students. The optimal time-use zone analysis indicated that the balanced 24-h movement behavior composition associated with the highest psychological wellbeing and the lowest anxiety symptoms consisted of 316 min/day of LPA, 68 min/day of MVPA, 510 min/day of SB, and 545 min/day of SLP. However, in school settings, students often have limited autonomy over their time use, making strict adherence to precise behavioral durations difficult. To improve flexibility and practical feasibility, this study proposed target ranges for each movement behavior based on the overlapping 24-h activity compositions corresponding to the top 10% of mental health outcomes: LPA (270–420 min/day), MVPA (50–110 min/day), SB (460–550 min/day), and SLP (470–610 min/day). It should be noted that these ranges are relatively broad; while this breadth supports individualized and flexible time allocation in practice, it also reflects estimation uncertainty in identifying exact optimal durations.

To date, only a limited number of studies have explored optimal 24-h movement behavior recommendations based on child and adolescent mental health (Dumuid et al., 2022b; Fairclough et al., 2023; Duncan et al., 2024). In China, empirical research on 24-h movement behavior recommendations has focused on adult mental health (Huang and Li, 2025; Li et al., 2026), leaving junior high school students under examined. Compared with existing 24-h movement behavior guidelines, the recommended MVPA duration of 68 min/day in this study is consistent with the guideline recommendation of at least 60 min/day of MVPA (Tremblay et al., 2016; Chen S. et al., 2022; Loo et al., 2022; Okely et al., 2022). Given the bounded nature of 24-h movement behaviors, behavior durations cannot increase or decrease indefinitely; therefore, considering the nonlinear association between MVPA and mental health, this study further proposes a target range of 50–110 min/day around the 68 min/day reference value. In addition, the recommended SLP duration of 545 min/day, approximately 9.1 h, is also consistent with the lower bound of the 9–11 h/day sleep in existing 24-h movement behavior guidelines (Tremblay et al., 2016; Loo et al., 2022; Okely et al., 2022). Existing guidelines do not provide explicit quantitative recommendations for total SB and LPA. Because MVPA accounts for a very small proportion of the day, the remaining waking time is primarily composed of LPA and SB, making it important to identify optimal durations for these two components. For these two under-specified behaviors, this study identified reference ranges for LPA (316 min/day; 270–420 min/day) and SB (510 min/day; 460–550 min/day) associated with more favorable mental health outcomes. A previous study reported that the necessary daily sedentary time for Chinese primary and secondary school students typically ranges from 420 to 600 min/day (Xie et al., 2024), which fully encompasses the SB range identified in this study and aligns with evidence of excessive sedentary time among Chinese children and adolescents (Wang et al., 2013). Therefore, the LPA and SB recommendations proposed in this study appear to be feasible within the school context. It is important to note, however, that the identified optimal time-use zone may vary across contexts. Regional differences in school schedules, academic demands, physical activity environments, and socioeconomic conditions may influence adolescents' 24-h movement behavior compositions. Therefore, although our research results provide a reference for time allocation for junior high school students, adjustments should be made in the future based on the local educational, geographical, and socio-economic characteristics when implementing.

It is noteworthy that, under the top 5% optimal mental health outcome thresholds, no overlapping region emerged between the 24-h movement behavior recommendation zones for maximizing psychological wellbeing and minimizing anxiety symptoms. Specifically, the top 5% of psychological wellbeing were associated with longer SLP duration, whereas the lowest 5% of anxiety were associated with longer LPA duration and shorter SB duration. This divergence suggests that psychological wellbeing and anxiety may have distinct time-allocation requirements within the daily 24-h movement composition. As a promotive positive psychological trait, psychological wellbeing may require sufficient sleep for maintenance. Supporting this interpretation, a recent meta-analysis of children and adolescents found that sleep had the strongest positive association with socio-emotional outcomes among all 24-h movement behaviors (Bourke et al., 2025). Previous studies have also consistently reported a negative association between SB and anxiety symptoms among children and adolescents. (de Faria et al., 2022; Kuzik et al., 2022; Bourke et al., 2025). LPA is accessible and achievable, helping children and adolescents relax, engage in casual social interactions, and regulate their emotions, while serving as a stepping stone toward MVPA (Telford et al., 2023). Higher LPA during waking hours may be associated with less sedentary time and greater opportunities for social interaction, which could partly explain its association with lower anxiety symptoms among junior high school students (Teychenne et al., 2015). In summary, promoting adolescent mental health requires a balanced 24-h movement behavior composition rather than isolated modifications of individual behaviors. Although the identified 24-h movement behavior profiles provide valuable reference points for daily time allocation, strict adherence to these targets may be challenging due to adolescents' limited autonomy over their daily schedules, particularly within school contexts. Therefore, further investigation into more flexible and adaptable time-allocation strategies is warranted.

### 4.2 Predictive associations between behavioral time reallocation and mental health

Beyond identifying recommended 24-h movement behavior targets, this study further explored whether flexible behavioral time-reallocation strategies could provide alternative options when adolescents are unable to strictly meet the absolute durations recommended by current 24-h movement behavior guidelines. To this end, this study utilized two substitution modes to examine the associations between simulated time reallocations among 24-h movement behaviors and mental health outcomes among junior high school students. Increasing SB while proportionally decreasing the other movement behaviors was associated with lower predicted psychological wellbeing scores and higher predicted anxiety scores. Conversely, increasing SLP while proportionally decreasing the remaining behaviors was associated with higher predicted psychological wellbeing scores and lower predicted anxiety scores. These findings are consistent with previous domestic and international evidence showing that sufficient SLP and reduced SB time are associated with better mental health (de Faria et al., 2022; Groves et al., 2024; Bourke et al., 2025; Huang and Li, 2025; He et al., 2026). The finding that increasing LPA while proportionally decreasing the remaining behaviors was primarily associated with higher psychological wellbeing adds to the limited evidence on this topic. An increasing body of research suggests that LPA may promote relaxation and contribute to enhanced wellbeing (van Woudenberg et al., 2020; Telford et al., 2023; Nyam et al., 2025). In contrast, increasing MVPA was primarily associated with lower anxiety. The one-to-one substitution results further suggest that small-scale adjustments in daily movement behavior may be associated with more favorable mental health outcomes. The most robust finding was that simulated reallocations of 15 min/day from SB to either LPA or SLP were associated with higher predicted psychological wellbeing scores and lower predicted anxiety scores, consistent with previous findings (de Faria et al., 2022; Huang and Li, 2025; Nyam et al., 2025). Notably, for anxiety symptoms, replacing 15 min/day of SB with MVPA was associated with a greater change in predicted anxiety scores, approximately 4 times that observed for replacing SB with LPA and 2 times that observed for replacing SB with SLP. This finding is consistent with previous studies of 24-h movement behaviors and mental health, which have also suggested that reallocating time toward MVPA is associated with the largest changes in predicted anxiety scores compared with reallocations toward other movement behaviors (de Faria et al., 2022; Wang et al., 2025).

Building upon the above substitution associations, this study further developed equivalent impact curves for 24-h movement behaviors to identify simulated time-allocation strategies associated with comparable changes in predicted mental health outcomes from a target-based perspective. For psychological wellbeing under the one-to-many substitution mode, the smallest magnitude of simulated time change was observed for SB reduction when corresponding to the same increase in predicted wellbeing scores (0.1 SD), followed by SLP increase and then LPA increase. Under the one-to-one model, direct replacement of SB with SLP corresponded to an equivalent change in predicted anxiety scores. In terms of time-allocation efficiency, the psychological wellbeing benefit associated with reducing one unit of SB was approximately 2.2 times the benefit associated with increasing LPA and approximately 1.2 times that associated with increasing SLP. For anxiety under the one-to-many model, the smallest time change required for the same improvement was an increase in MVPA, followed by a reduction in SB and then an increase in SLP. Under the one-to-one model, directly replacing SB with SLP produced equivalent emotional benefits. The magnitude of change in predicted anxiety scores associated with a one-unit relative increase in MVPA was approximately 1.5 times that associated with a one-unit relative reduction in SB and approximately 1.7 times that associated with a one-unit relative increase in SLP.

Prior isotemporal substitution studies have suggested that reallocating time toward MVPA is associated with relatively larger changes in predicted obesity indicators and health-related quality of life among children and adolescents (Talarico and Janssen, 2018; Ng et al., 2021). However, because the present study focused on mental health outcomes and considered the baseline time proportions of each movement behavior, relative reductions in SB showed the strongest correlational magnitude with favorable mental health profiles per unit of simulated time change. For example, the magnitude of simulated SB reduction corresponding to a 0.1-SD difference in psychological wellbeing and anxiety scores accounted for only approximately 4% of the sample's sedentary compositional mean. In concrete terms, a 0.1 SD shift corresponds to an approximate raw score difference of 1.5 points on the EPOCH psychological wellbeing scale and 2.2 points on the MASC-C anxiety scale, whereas a 0.2 SD shift corresponds to differences of approximately 3.1 and 4.4 points, respectively. The corresponding behavioral reallocations of approximately 20–60 min per day fall within a realistic range of adjustments that may be feasible in adolescents' daily routines. Because current 24-h movement behavior guidelines for children and adolescents do not provide specific limits for total SB duration, the equivalent-impact findings of this study suggest that future guideline optimization may explore small, accessible compositional reallocations of total SB as a potential direction. Low-threshold approaches such as sedentary breaks (Ma et al., 2020, 2026) and short bouts of accumulated exercise (Yin et al., 2025) have been proposed to interrupt prolonged sedentary periods, but their actual effects on adolescent mental health remain to be verified by intervention studies.

The equivalent impact curves further indicated that no single, unique time-allocation pattern was exclusively associated with favorable mental health outcomes in daily life. Instead, multiple movement behavior reallocation patterns may correspond to comparable levels of psychological wellbeing or anxiety outcomes of ± 0.1 or ± 0.2 SD. These findings provide preliminary correlational evidence that may inform future intervention research by highlighting the potential value of flexible and individualized 24-h movement behavior optimization approaches rather than rigid one-size-fits-all guidelines for child and adolescent mental health.

### 4.3 Implications for flexible and individualized interventions

The equivalent impacts of 24-h movement behaviors identified in this study provide important practical implications for school-based physical activity promotion and mental health initiatives. By demonstrating multiple behavioral pathways associated with comparable predicted mental health outcomes, these findings offer a new perspective for addressing the generally low adherence to current 24-h movement behavior guidelines worldwide. Children and adolescents with the same health status may have substantially different 24-h movement behavior time-allocation patterns (Ng et al., 2021). Therefore, alternative 24-h movement behavior configurations may correspond to comparable predicted mental health profiles rather than being tied to a single fixed allocation pattern. Instead, it can be flexibly adapted to the individual preferences and lifestyle constraints, thereby increasing autonomy and making lifestyle interventions more versatile. Global compliance with 24-h movement guidelines among children and adolescents remains low (Hossian et al., 2025), and recent evidence indicates that only 4.91% of Chinese children and adolescents meet all three guidelines (Feng and Lou, 2026). In this context, the equivalent-impact framework provides a potential pathway for intervention. Even when individuals cannot fully meet the absolute durations recommended by guidelines, movement behavior combinations corresponding to comparable mental health profiles may exist, such as patterns with lower SB and higher SLP when higher MVPA is not achievable. Furthermore, allowing individuals to take responsibility and make choices in designing behavior-change strategies may be critical for long-term compliance and intervention success (Burgess et al., 2017). These correlational findings suggest that future movement behavior intervention research may explore shifting from absolute targets toward flexible time-allocation schemes to test whether more adaptable options can support sustained behavior change and more favorable mental health outcomes.

Exploring equivalent impacts provides new opportunities for flexible, person-centered behavioral interventions by accommodating individual preferences and real-life constraints among youth. Whether through a one-to-many framework (increasing a target behavior via proportional reallocation across remaining behaviors) or a one-to-one model (directly replacing one behavior with another), these approaches may help educators, clinicians, and adolescents develop tailored lifestyle modification strategies that are both feasible and contextually appropriate.

### 4.4 Strengths, limitations, and future directions

This study has several strengths. First, it used validated, reliable, and widely applied instruments to measure both exposures and outcomes. Second, the compositional data analysis framework addressed the constant-sum constraints, closure properties, and multicollinearity that limit traditional approaches to analyzing time-use data across movement behaviors. Third, to our knowledge, this study is the first to construct equivalent impact curves for mental health outcomes among Chinese adolescents, providing new insights into the associations of time reallocation across 24-h movement behaviors with psychological outcomes. Nevertheless, limitations should be acknowledged. The cross-sectional design of this study precludes the determination of causal directions between 24-h movement behaviors and mental health. The observed associations between greater sedentary time and poorer mental health may reflect bidirectional relationships or potential reverse causality; for example, adolescents with poorer mental health status may be more likely to engage in prolonged sedentary behavior, lower levels of physical activity, and suboptimal sleep patterns, rather than sedentary behavior itself leading to poorer mental health. This possibility cannot be excluded. Future studies using longitudinal cohort designs or experimental interventions are needed to clarify temporal relationships and determine whether changes in movement behaviors influence mental health outcomes. In addition, 24-h movement behaviors were assessed as aggregated durations without distinguishing their specific contexts or domains. In real-world settings, physical activity performed in different contexts may have distinct associations with mental health, and the mental health correlates of sedentary behavior may vary depending on whether it occurs during structured academic tasks, recreational screen use, or social interactions. Future research could integrate device-based monitoring with ecological momentary assessment (EMA) or domain-specific accelerometry approaches to capture these contextual nuances and further elucidate domain-specific 24-h movement behavior patterns. Third, this study relied on a convenience sampling approach, with participants recruited exclusively from four junior high schools in Chengdu. Due to incomplete data, the baseline characteristics of excluded participants could not be systematically documented, nor could formal analyses of non-response or attrition bias be conducted. Consequently, the identified 24-h movement behavior patterns and optimal time-use estimates may not be directly generalizable to adolescents in rural areas, other regions of China, or populations with diverse socioeconomic and educational backgrounds. Given the marked regional disparities in school schedules, academic pressures, physical activity environments, and sports infrastructure across China, future multicenter studies utilizing larger, nationally representative cohorts are warranted to confirm the broader applicability and generalizability of these findings. Furthermore, it is worth noting that the optimal time-use zones identified in this study exhibited relatively wide ranges for certain movement behavior components. For example, the LPA range associated with the top 5% of psychological wellbeing scores spanned from 120 to 300 min/day. These broad intervals reflect the uncertainty inherent in estimating precise time-use patterns for optimal mental health outcomes and suggest that multiple time-use configurations may confer similar benefits. Future studies with larger sample sizes and more refined exposure assessments are warranted to improve the precision of these estimates.

## 5 Conclusion

Using a compositional data analysis framework, this study identified optimal 24-h movement behavior compositions associated with better mental health and evaluated the equivalent associations of daily time reallocations from a target-based perspective among Chinese junior high school students in Chengdu. By identifying the intersection of the optimal outcome zones, this study derived a balanced 24-h movement behavior composition associated with better psychological wellbeing and fewer anxiety symptoms: LPA, 316 min/day (270–420); MVPA, 68 min/day (50–110); SB, 510 min/day (460–550); and SLP, 545 min/day (470–610). These movement behavior ranges can be interpreted as preliminary reference values. The equivalent impact curves further demonstrated that comparable mental health profiles may correspond to multiple distinct time-allocation combinations. Considering the baseline time proportions of each behavior, relative reductions in SB showed the strongest magnitude of association with favorable mental health outcomes per unit of simulated time change. Notably, the magnitude of simulated SB reduction corresponding to a 0.1-SD difference in psychological wellbeing and anxiety scores accounted for only approximately 4% of the sample's daily baseline sedentary time. As optimal time-use compositions may vary across regional and contextual settings, these findings provide preliminary correlational evidence to guide future investigations of 24-h movement behaviors and adolescent mental health in urban southwest China, although further validation in diverse populations is warranted. Overall, these findings suggest that exploring flexible time-allocation approaches that accommodate individual preferences and contextual characteristics may represent a promising direction for future research on 24-h movement behaviors and adolescent mental health.

## Data Availability

The anonymized raw data supporting the conclusions of this article will be made available by the corresponding author upon reasonable request, subject to participant privacy and institutional data protection requirements.
