# Supplementary material for: Recommended 24-h movement behavior patterns and equivalent impacts on mental health among Chinese adolescents: a compositional data analysis
Source: Front Psychol. 2026 Sep 9;17:1936732. doi: 10.3389/fpsyg.2026.1936732 (PMC13597398; doi:10.3389/fpsyg.2026.1936732)
Supplement: Supplementary file 1 [file Data_Sheet_1.pdf]

Supplementary Table S1. Linear Regression Model Diagnostics for 24-h Movement Behavior Compositions and Mental Health Outcomes

Diagnostic Evaluation Domain	Statistical Metric / Test	Psychological Wellbeing Score model	Anxiety Score model
Multicollinearity	Adjusted VIF ($GVIF^{1/(2×Df)}$) – ilr coordinates	1.377−1.789	1.377−1.789
	Adjusted VIF ($GVIF^{1/(2×Df)}$) – Covariates	1.043−1.709	1.043−1.709
Influential Outliers	Maximum Cook's Distance (Cook's *D*)	0.049	0.044
Residual Autocorrelation	Durbin-Watson Statistic (*d*)	1.924 (*P*=0.352)	1.925 (*P*=0.418)
Residual Normality	Shapiro-Wilk Test Statistic (*W*)	0.991 (*P*=0.041)	0.989 (*P*=0.013)
Homoscedasticity	Breusch-Pagan Test Statistic (BP)	42.616 (*P*=0.001)	34.384 (*P*=0.017)

Abbreviations: ilr, isometric log-ratio; VIF, Variance Inflation Factor; GVIF, Generalized Variance Inflation Factor.

Diagnostic criteria:

- No problematic multicollinearity was detected, as all adjusted GVIF values, expressed as $GVIF^{(1/(2×Df))}$, were well below the conventional threshold of 5.0.
- No highly influential outliers were identified, with all Cook's *D* values far below the 0.50 threshold.
- Residuals showed no significant autocorrelation, with Durbin–Watson statistics close to 2 and all *P* values > 0.05.
- The statistically significant results of the Shapiro–Wilk and Breusch–Pagan tests were interpreted jointly with diagnostic plots. Given the known sensitivity of these tests to large sample sizes (N=319), visual inspection suggested approximately normal residual distributions and only mild heteroscedasticity, suggesting that the regression estimates were unlikely to be substantially affected.
- The linearity assumption was verified via residual-versus-fitted plots (Supplementary Figures S1–S2).

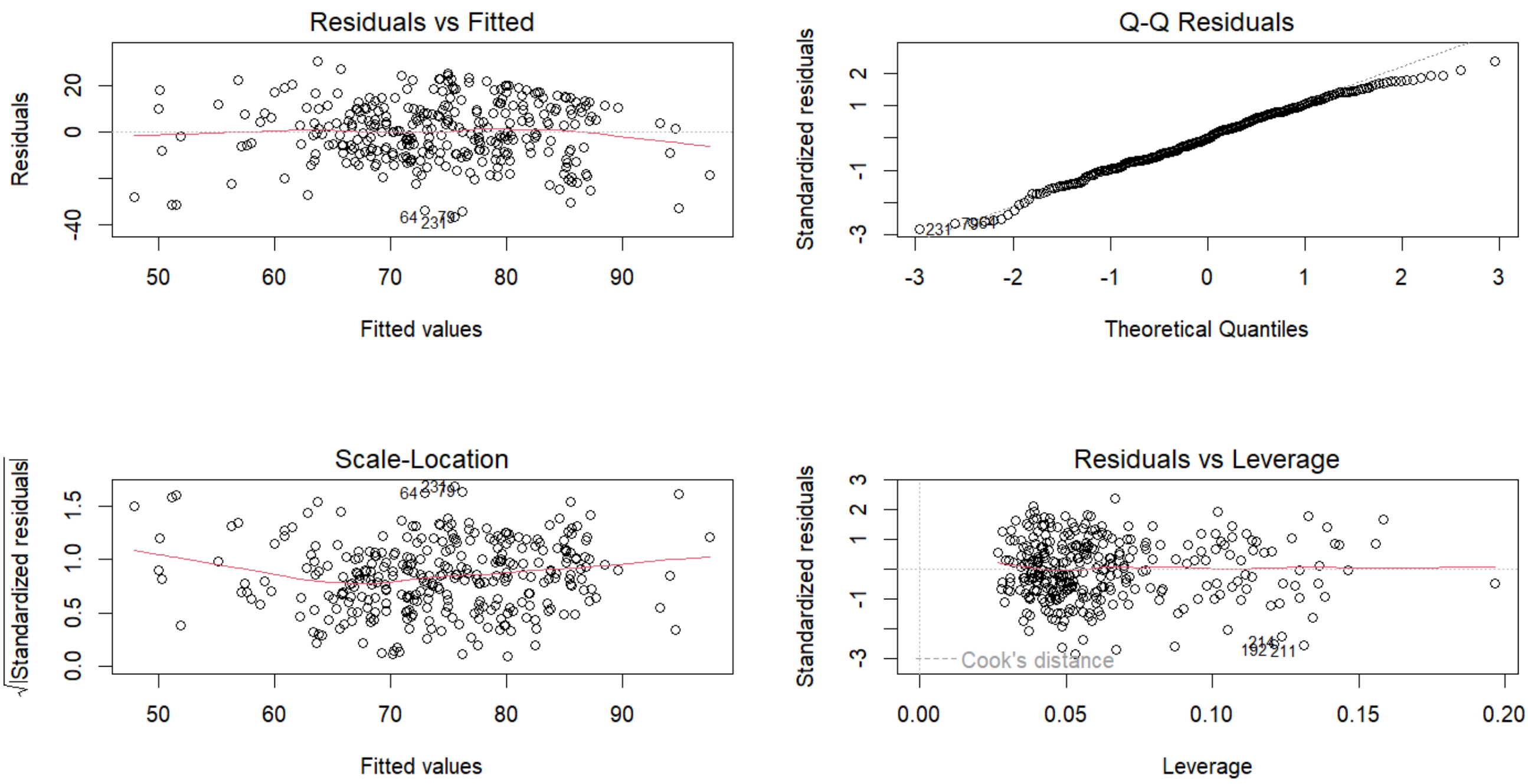

Supplementary Figure S1. Linear regression diagnostics for the psychological wellbeing model: residual analysis and influence point assessment.

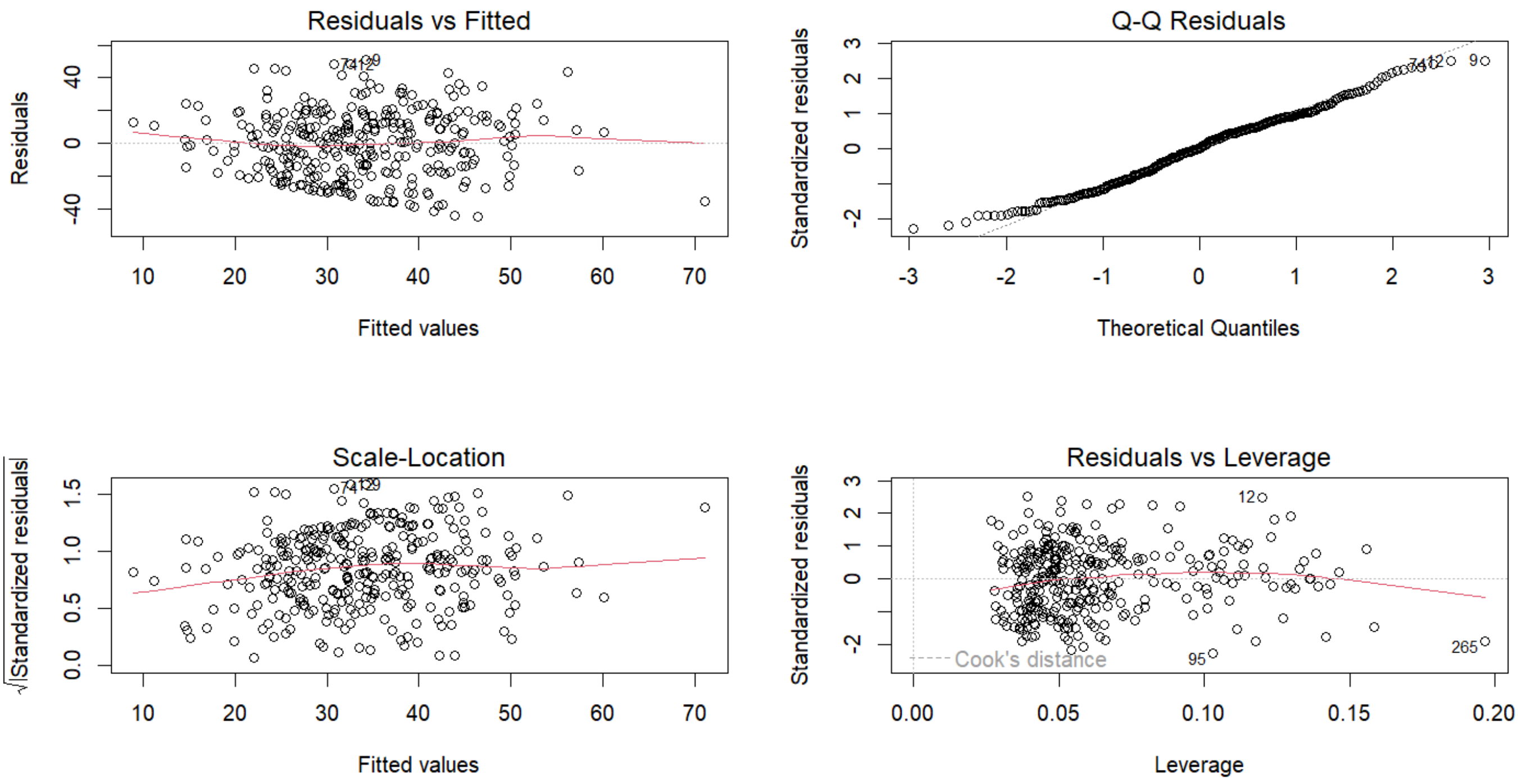

Supplementary Figure S2. Linear regression diagnostics for the anxiety score model: residual analysis and influence point assessment.
