# Supplementary material for: Recommended 24-h movement behavior patterns and equivalent impacts on mental health among Chinese adolescents: a compositional data analysis
Source: Front Psychol. 2026 Sep 9;17:1936732. doi: 10.3389/fpsyg.2026.1936732 (PMC13597398; doi:10.3389/fpsyg.2026.1936732)
Supplement: Supplementary file 2 [file Data_Sheet_2.pdf]

Supplementary Text S1

1. Mathematical Formulation and Practical Numerical Illustration of Compensation Factor S

1.1 Mathematical Derivation

Consider a 24-h movement behavior composition represented as a four-part vector:

$$\mathbf{X} = [X_{\text{LPA}}, X_{\text{MVPA}}, X_{\text{SB}}, X_{\text{SLP}}]^T$$

The composition is strictly subject to the constant-sum (closure) constraint:

$$\sum_{i=1}^{4} X_i = 1440 \text{ min/day}$$

Under the one-to-many isoproportional substitution model, when a single target behavior X_k (e.g., X_{LPA}) is reallocated by a duration change of ΔX_k, the remaining three non-target behaviors X_j ($j \neq k$) adjust proportionally relative to their baseline compositional means:

$$X_j^{\text{new}} = X_j^{\text{baseline}} \times (1 - S)$$

To ensure that the newly generated composition $\mathbf{X}^{\text{new}}$ satisfies the constant-sum budget (1440 min/day):

$$\Delta X_k + \sum_{j \neq k} \left(X_j^{\text{new}} - X_j^{\text{baseline}} \right) = 0$$

Substituting $X_j^{\text{new}} = X_j^{\text{baseline}} \times (1 - S)$ into the closure constraint equation yields:

$$\Delta X_k + \sum_{j \neq k} \left[X_j^{\text{baseline}} \times (1 - S) - X_j^{\text{baseline}} \right] = 0 \Longrightarrow \Delta X_k - S \sum_{j \neq k} X_j^{\text{baseline}} = 0$$

Since the sum of the non-target baseline behaviors equals the total daily duration minus the baseline duration of the target behavior ($\sum_{j \neq k} X_j^{\text{baseline}} = 1440 - X_k^{\text{baseline}}$), the compensation factor S is explicitly solved as:

$$S = \frac{\Delta X_k}{1440 - X_k^{\text{baseline}}}$$

1.2 Numerical Worked Example

To illustrate this calculation intuitively, we apply the empirical compositional geometric means from the study sample as the baseline time allocation:

- **LPA** = 203.22 min/day
- **MVPA** = 41.14 min/day
- **SB** = 695.34 min/day
- **SLP** = 500.29 min/day
- **Total Budget** = 1440.00 min/day

Scenario: Assume LPA increases by 10% of its baseline compositional mean ($\Delta X_{\text{LPA}} = +20.32$ min/day).

- **Step 1: Calculate the total baseline duration of non-target behaviors.**

The sum of MVPA, SB, and SLP equals:

$$1440 - 203.22 = 1236.78\,\text{min}$$

- **Step 2: Calculate the relative proportions of non-target behaviors.**

Within the remaining $1236.78\,\text{min}$, the proportion of each non-target behavior is:

  - MVPA: $41.14/1236.78 \approx 3.33\%$
  - SB: $695.34/1236.78 \approx 56.22\%$
  - SLP: $500.29/1236.78 \approx 40.45\%$

- **Step 3: Proportionally deduct the added $20.32\,\text{min}$ from non-target behaviors.**

To maintain the $1440\,\text{min}$ total budget, the $+20.32\,\text{min}$ increase in LPA is offset according to these relative percentages:

  - MVPA reduction $= 20.32 \times 3.33\% = 0.68\,\text{min} \Longrightarrow$ New MVPA $= 41.14 - 0.68 = 40.46\,\text{min}$
  - SB reduction $= 20.32 \times 56.22\% = 11.42\,\text{min} \Longrightarrow$ New SB $= 695.34 - 11.42 = 683.92\,\text{min}$
  - SLP reduction $= 20.32 \times 40.45\% = 8.22\,\text{min} \Longrightarrow$ New SLP $= 500.29 - 8.22 = 492.07\,\text{min}$

- **Step 4: Verify via the Compensation Factor S Formula.**

$$S = \frac{20.32}{1440 - 203.22} = \frac{20.32}{1236.78} \approx 0.01643 \quad (1.643\%)$$

Multiplying non-target baseline values by $(1 - S) = 1 - 0.01643 = 0.98357$:

  - New MVPA $= 41.14 \times 0.98357 = 40.46\,\text{min}$
  - New SB $= 695.34 \times 0.98357 = 683.92\,\text{min}$
  - New SLP $= 500.29 \times 0.98357 = 492.07\,\text{min}$

Both methods produce identical results.

2. Step-Wise Grid Search Algorithmic Procedure for Equivalent Impact Curves

The equivalent impact curves (iso-effect contours) mapping predefined outcome changes ($\Delta\hat{Y}_{\text{target}} \in \{+0.2\,\text{SD}, +0.1\,\text{SD}, -0.1\,\text{SD}, -0.2\,\text{SD}\}$) under the one-to-many isoproportional substitution model were generated using a 4-step grid search algorithm in R:

Step 1: Baseline Reference Profile and Prediction Setting

- The reference 24-h movement composition $\mathbf{X}_{\text{base}}$ was assigned to the sample compositional geometric mean:

$$\mathbf{X}_{\text{base}} = \left[X_{\text{SLP}}^{\text{base}}, X_{\text{SB}}^{\text{base}}, X_{\text{LPA}}^{\text{base}}, X_{\text{MVPA}}^{\text{base}}\right]^T$$

- Continuous covariates $\mathbf{Z}_{\text{cont}}$ (e.g., age, BMI) were set to sample arithmetic means, and categorical covariates $\mathbf{Z}_{\text{cat}}$ (e.g., gender, grade, resident status, parental education, household income) were set to sample modes to define a standardized reference profile $\mathbf{Z}_{\text{ref}}$.
- The baseline isometric log-ratio coordinates ilr_{base} were calculated, and the baseline predicted outcome score was established as:

$$\hat{Y}_{\text{base}} = f(ilr_{\text{base}}, \mathbf{Z}_{\text{ref}})$$

Step 2: Fine Grid Sequence Generation

- For a given target behavior X_k ($k \in \{\text{SLP,SB,LPA,MVPA}\}$), a sequence of potential duration changes ΔX_k was constructed across a fine grid ranging from $-150\,\text{min/day}$ to $+150\,\text{min/day}$ at a step size of $\delta = 0.1\,\text{min}$:

$$\Delta X_k \in \mathcal{G} = \{-150.0, -149.9, \ldots, 0.0, \ldots, +149.9, +150.0\}$$

Step 3: Dynamic Proportional Reallocation and Score Evaluation

- At each grid step $\Delta X_k \in \mathcal{G}$, the updated duration of the target behavior was defined as $X_k^{\text{new}} = X_k^{\text{base}} + \Delta X_k$.
- The remaining non-target behaviors X_j ($j \neq k$) were proportionally reallocated to maintain the $1440\,\text{min/day}$ daily budget using the dynamic proportion factor $\text{prop}(\Delta X_k)$:

$$X_j^{\text{new}}(\Delta X_k) = X_j^{\text{base}} \times \text{prop}(\Delta X_k) = X_j^{\text{base}} \times \left(\frac{1440 - X_k^{\text{base}} - \Delta X_k}{1440 - X_k^{\text{base}}} \right)$$

- Grid compositions resulting in any non-positive duration ($X_i^{\text{new}} \leq 0$) were truncated.
- Valid updated compositions $\mathbf{X}^{\text{new}}(\Delta X_k)$ were transformed into $ilr_{\text{new}}(\Delta X_k)$ coordinates and entered into the fitted linear model to evaluate the predicted score difference relative to baseline:

$$\Delta \hat{Y}(\Delta X_k) = f(ilr_{\text{new}}(\Delta X_k), \mathbf{Z}_{\text{ref}}) - \hat{Y}_{\text{base}}$$

Step 4: Iso-Effect Target Matching and 2D Pairwise Curve Construction

- For each target standardized effect size $\Delta \hat{Y}_{\text{target}} \in \{\pm 0.1\,\text{SD}, \pm 0.2\,\text{SD}\}$, the required duration shift ΔX_k^* was extracted by minimizing the absolute prediction error over the grid $\mathcal{G}$:

$$\Delta X_k^* = \arg \min_{\Delta X_k \in \mathcal{G}} \left| \Delta \hat{Y}(\Delta X_k) - \Delta \hat{Y}_{\text{target}} \right|$$

- To construct the 2D equivalent impact curves for any pair of movement behaviors (e.g., Behavior A on the x-axis and Behavior B on the y-axis), the extracted duration changes achieving the identical target effect size were paired as coordinates:

$$\mathbf{P}_{\text{target}} = (\Delta X_A^*, \Delta X_B^*)$$

- Linear trajectories connecting these target coordinates $(\Delta X_A^*, \Delta X_B^*)$ through the origin $(0,0)$ were rendered to visualize the equivalent reallocation trade-offs.

3. Code Availability and Reproducibility

To ensure complete computational transparency and reproducibility, the executable R script implementing this step-wise grid search algorithm, model diagnostics, and ggplot figure generation is publicly archived on the Open Science Framework (OSF) repository:

URL: https://osf.io/24cqa/
